# Association of Kidney Function With Dementia and Structural Brain Differences: A Large Population-Based Cohort Study

**DOI:** 10.1093/gerona/glad192

**Published:** 2023-08-14

**Authors:** Shuqi Wang, Jiao Wang, Jie Guo, Abigail Dove, Hong Xu, Xiuying Qi, Weili Xu

**Affiliations:** Department of Epidemiology and Biostatistics, School of Public Health, Tianjin Medical University, Tianjin, China; Department of Epidemiology, College of Preventive Medicine, the Army Medical University (Third Military Medical University), Chongqing, China; Department of Neurobiology, Care Sciences and Society, Karolinska Institutet, Stockholm, Sweden; Department of Neurobiology, Care Sciences and Society, Karolinska Institutet, Stockholm, Sweden; Department of Neurobiology, Care Sciences and Society, Karolinska Institutet, Stockholm, Sweden; Department of Epidemiology and Biostatistics, School of Public Health, Tianjin Medical University, Tianjin, China; Department of Epidemiology and Biostatistics, School of Public Health, Tianjin Medical University, Tianjin, China; Department of Neurobiology, Care Sciences and Society, Karolinska Institutet, Stockholm, Sweden; (Medical Sciences Section)

**Keywords:** Brain volume, Dementia, Kidney function, Magnetic resonance imaging, Population-based cohort study

## Abstract

**Background:**

The association between kidney function and dementia risk and the mechanisms underlying this relationship remain unclear.

**Methods:**

Within the UK Biobank, 191 970 dementia-free participants aged ≥60 (mean age: 64.1 ± 2.9 years) were followed for 16 years to detect incident dementia. Serum creatinine and Cystatin C were measured at baseline to calculate estimated glomerular filtration rate (eGFR, mL/min/1.73 m^2^). Kidney function was categorized as normal (eGFR ≥ 90), mildly impaired (60 ≤ eGFR < 90), or moderately to severely impaired (eGFR < 60). Dementia was assessed based on self-reported medical history and medical records. During the follow-up, a subsample of 12 637 participants underwent brain MRI scans. Volumes of total brain, gray matter, white matter, hippocampus, and white matter hyperintensities were assessed.

**Results:**

Over the follow-up, 5 327 (2.8%) participants developed dementia. Compared to normal kidney function, there was an increased risk of dementia with moderate to severely impaired kidney function (hazard ratio = 1.53, 95% confidence interval [CI]: 1.32–1.76) but not mildly impaired kidney function. In Laplace regression, dementia onset among people with moderate to severely impaired kidney function occurred 1.53 (95% CI: 0.98–2.08) years earlier than those with normal kidney function. Moderate to severely impaired kidney function was related to significantly lower gray matter volume (β = −0.11, 95% CI: −0.19 to −0.03), but not to other brain magnetic resonance imaging measures.

**Conclusions:**

Impaired kidney function is associated with about 50% increased risk of dementia and anticipates dementia onset by more than 1.5 years. Brain neurodegeneration may underlie the kidney function–dementia association.

Chronic kidney disease (CKD) affects about 12% of the global population (1) and has continued to rise in rank among the leading causes of death for older adults (2). Kidney function declines with aging. Estimated glomerular filtration rate (eGFR) based on serum creatinine and Cystatin C is a widely used measurement of kidney function in clinical practice (3). Kidney function is considered impaired when estimated eGFR is <90 mL/min/1.73 m^2^ (3). Impaired kidney function can progress to CKD (eGFR < 60 mL/min/1.73 m^2^) and eventually end-stage renal disease (eGFR < 15 mL/min/1.73 m^2^) (3).

Impaired kidney function, even in its early stages, has been associated with an increased risk of cognitive impairment (4). A previous cross-sectional study based on UK Biobank data reported that people with eGFR < 60 mL/min/1.73 m^2^ had worse performance on cognitive tests reflecting verbal/numeric reasoning and reaction time compared to those with eGFR ≥ 60 mL/min/1.73 m^2^ (5). The relationship between impaired kidney function and dementia is unclear, with most previous cohort studies reporting a significantly increased risk of dementia (6–12) in relation to impaired kidney function, as well as Alzheimer’s disease (AD) (8–10) and vascular dementia (9–12), whereas others reported no such association (13–15). Open questions remain regarding whether and by what mechanisms impaired kidney function may affect dementia onset among older adults.

Magnetic resonance imaging (MRI) markers can indicate cerebral microvascular and neurodegenerative pathologies in the brain (16). Early detection of brain structural abnormalities provides an opportunity to assess possible mechanisms underlying the development of dementia. Some previous studies have explored the relationship between kidney function and brain MRI parameters, but these have been based on relatively small sample sizes with inconsistent results (17–24). Some studies have linked impaired kidney function to a greater burden of white matter hyperintensities (WMH) (17–19) and decreased white matter (20) and gray matter (21) volume, while others found no such associations (22–24).

In the present study, we aimed to (1) investigate the association between kidney function and the risk of dementia, including AD and vascular dementia, and (2) explore the relationship between kidney function and regional brain volumes on MRI using data from the UK Biobank, a large population-based cohort with an embedded neuroimaging study.

## Method

### Study Population

The study population was derived from the UK Biobank. From 2006 to 2010, 502 412 participants from 22 assessment centers across the United Kingdom were recruited in the baseline survey. Between 2014 and 2020, a subsample of 42 806 participants underwent brain MRI scans (25).

**Figure 1.**
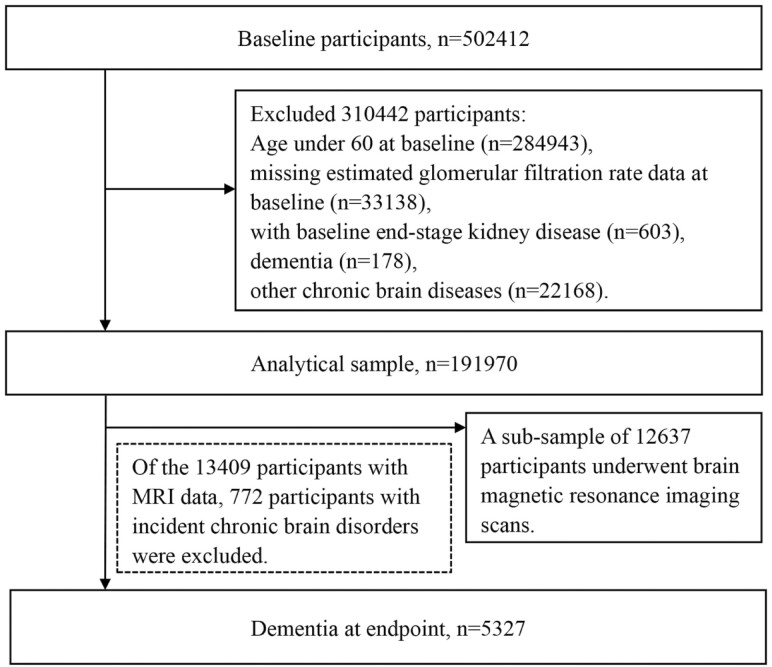
Flowchart of the study population.

Of 502 412 participants, 217 469 were in older age (≥60 years) at baseline. From this group, we excluded 603 with end-stage kidney disease, 178 with prevalent dementia, 22 168 with other chronic brain disorders besides dementia (including stroke, Parkinson’s disease, brain hemorrhage, transient ischemic attack, etc.; see Supplementary Table 1 for more information). Additionally, 33 138 individuals were excluded due to missing information on eGFR. This left a study population of 191 970 dementia-free individuals for the analysis of the association between kidney function and dementia. Of these, 13 409 participants underwent brain MRI. After excluding 772 participants who developed incident chronic brain disorders between baseline and the MRI scan, 12 637 individuals were included in the analysis of the association between kidney function and regional brain volumes (Figure 1).

The UK Biobank study received ethical approval from the North West Multi-Centre Research Ethics Committee (Ref 11/NW/0382). All participants provided written informed consent and all data used in this study were obtained from the UK Biobank (http://www.ukbiobank.ac.uk) through application 67048.

### Data Collection

Participants underwent comprehensive physical and clinical examinations at baseline and provided information on demographic characteristics, socioeconomic status, and lifestyle factors through a computerized touchscreen questionnaire.

Race was dichotomized as White or non-White. The White category included people with British, Irish, or any other White background, and the non-White category included people with mixed, Asian, or Black backgrounds. Socioeconomic status was measured using the Townsend Deprivation Index (26). Education level was categorized as university or non-university. Body mass index (BMI) was calculated as weight (kg) divided by squared height (m^2^). Smoking and alcohol consumption was categorized as never, previous, or current. Regular physical activity was defined as at least 150 minutes of moderate activity per week, 75 minutes of vigorous activity per week, or an equivalent combination (27). The social connection was evaluated based on responses to the question “How often do you visit friends or family or have them visit you?” and classified as rich (“almost daily,” “2-4 times a week,” “about once a week,” and “about once a month”) or poor (“once every few months,” “never or almost never,” and “no friends/family outside household”) according to the median.

Baseline diabetes was diagnosed based on glycated hemoglobin A1c (≥6.5%), fasting plasma glucose (≥126 mg/dl), random blood glucose (≥200 mg/dL), use of diabetes medication, self-reported history of diabetes, or medical records (including primary care, inpatient care, and the death registry) (28). Baseline hypertension was assessed via measured systolic/diastolic blood pressure ≥ 140/90 mm Hg, use of antihypertensive drugs, self-reported history of hypertension, or medical records. Baseline heart disease (including myocardial infarction, angina, congestive heart failure, and atrial fibrillation) and end-stage renal disease were assessed based on self-reported medical history and medical records. All diagnoses are recorded according to the 9th and 10th versions of the International Classification of Diseases (ICD).

The apolipoprotein E (*APOE*) gene was genotyped and dichotomized as ε4 allele carriers versus non-carriers. Urine samples were collected from all participants at baseline, and urinary albumin/creatinine ratio (uACR) was estimated by dividing urinary albumin by urinary creatinine and was dichotomized as normal (<30 mg/g) or abnormal (≥30 mg/g). More details are shown in Supplementary Table 2.

### Assessment of Kidney Function

As part of the UK Biobank Biomarker Project, blood samples were collected from participants at baseline, and biomarkers were measured using validated assays (29,30). Serum creatinine was measured by enzymatic analysis on a Beckman Coulter AU5800. Serum cystatin C was measured by latex-enhanced immunoturbidimetric analysis on a Siemens ADVIA 1800. The estimated eGFR (mL/min/1.73 m^2^) was calculated using the Chronic Kidney Disease Epidemiology Collaboration (CKD-EPI) 2012 formula with creatinine and Cystatin C (31), accounting for age, ethnicity, and sex. This equation is recommended by Kidney Disease: Improving Global Outcomes work group for all adults (3) and is widely used in the United Kingdom (32). In sensitivity analyses, we used 3 alternative equations: the CKD-EPI 2009 Creatinine Equation (only using creatinine) (31), the CKD-EPI 2021 Race-Free (creatinine and Cystatin C) Equation (33), and the European Kidney Function Consortium (EKFC) Equation (34).

According to the eGFR value, kidney function was categorized as normal (>90 mL/min/1.73 m^2^, as reference), mildly impaired (60–90 mL/min/1.73 m^2^), or moderately to severely impaired (<60 mL/min/1.73 m^2^) (3).

### Dementia Diagnosis

Prevalent and incident cases of dementia (including AD and vascular dementia) were identified through self-reported medical history of dementia, data from the inpatient registry, and information from the death registry. The date of dementia diagnosis was set as the earliest date of the record regardless of the source used. Detailed information on the ICD-9 and ICD-10 codes used to identify dementia are shown in Supplementary Table 3.

### Brain MRI Acquisition and Processing

Magnetic resonance imaging (MRI) data were collected from 4 imaging centers located in Cheadle, Reading, Newcastle, and Bristol. Information about image acquisition and processing is available at the UK Biobank website in the brain scan protocol and brain imaging documentation (35,36). Briefly, participants were scanned with a Siemens Skyra 3T scanner with a standard Siemens 32-channel head coil. T1-weighted imaging (resolution: 1.0 × 1.0 × 1.0 mm; field-of-view: 208 × 256 × 256 matrix) and T2 FLAIR imaging (resolution: 1.05 × 1.0 × 1.0 mm; field-of-view: 192 × 256 × 256 matrix) were performed to provide volumes of brain tissues and structures. Summary measures of brain structure were generated by an image-processing pipeline developed and run on behalf of the UK Biobank, using publicly available image-processing tools (the FMRIB Software Library, version 5.0.10 and FreeSurfer, version 6.0) (37).

In this study, the volumes (in cubic millimeters) of total brain, gray matter, white matter, hippocampus, and WMH were assessed. Extreme outlying data points (further than ±4 standard deviations [*SD*] from the mean) were excluded (0.002% of the total imaging-derived phenotype data analyzed). All MRI parameters were converted to *z*-scores, and WMH volume was log-transformed due to its skewed distribution.

### Statistical Analysis

Baseline characteristics of the study population were compared by level of kidney function using Chi-square tests for categorical variables and one-way analysis of variance or Wilcoxon rank-sum tests for continuous variables.

Cox proportional hazards models were used to estimate the hazard ratios (HRs) and 95% confidence intervals (CIs) for the risk of all-cause dementia, AD, and vascular dementia in relation to kidney function (as both a continuous and a categorical variable). Follow-up time was used as the timescale and calculated as the time from study entry until dementia diagnosis, death, or the final examination, whichever occurred first. The proportional hazard assumption was tested using Schoenfeld residuals regressed against follow-up time; no violations were observed. Next, Laplace regression models were used to estimate the 10th percentile differences (PDs) in time (years) of all-cause dementia, AD, and vascular dementia onset across different levels of kidney function. Age, sex, race, education, socioeconomic status, BMI, smoking status, alcohol consumption, physical activity, social connection, diabetes, hypertension, heart disease, and *APOE* genotype were considered potential confounders (the numbers of missing data were shown in Table 1).

**Table 1.** Characteristics of the Study Population by Kidney Function Level (*n* = 191 970).

Characteristics	Kidney Function			*p* Value
	Normal (*N* = 60 837)	Mildly impaired (*N* = 122 463)	Moderately to severely impaired (*N* = 8 670)	
Creatinine, mg/dL	0.72 ± 0.12	0.86 ± 0.15	1.22 ± 0.38	<.001
Cystatin C, mg/L	0.81 ± 0.07	0.99 ± 0.11	1.40 ± 0.30	<.001
eGFR, mL/min/1.73 m^2^	96.89 ± 5.10	78.64 ± 7.58	51.60 ± 8.32	<.001
Age at baseline, yrs	63.38 ± 2.69	64.39 ± 2.85	65.41 ± 2.81	<.001
Female	32 509 (53.44%)	64 648 (52.79%)	4 689 (54.08%)	<.001
Race (White)	56 350 (92.68%)	114 145 (93.30%)	8 083 (93.35%)	<.001
Socioeconomic status	−2.52 (−3.84, −0.27)	−2.37 (−3.73, 0.06)	−1.83 (−3.44, 1.24)	.200
Education (university)	18 410 (30.61%)	30 298 (25.13%)	1 442 (16.96%)	<.001
BMI, kg/m^2^	26.28 ± 3.97	28.00 ± 4.52	30.28 ± 5.57	<.001
Smoking status				<.001
Never	31 114 (51.40%)	61 016 (50.13%)	3 851 (44.80%)	
Previous	25 514 (42.15%)	50 167 (41.22%)	3 786 (44.04%)	
Current	3 903 (6.45%)	10 532 (8.65%)	959 (11.16%)	
Alcohol consumption				<.001
Never	56 889 (93.67%)	111 442 (91.20%)	7 358 (85.15%)	
Previous	1 814 (2.99%)	4 535 (3.71%)	551 (6.38%)	
Current	2 031 (3.34%)	6 219 (5.09%)	732 (8.47%)	
Regular physical activity	41 363 (74.18%)	77 885 (71.18%)	4 390 (60.35%)	<.001
Rich social activity	29 294 (48.45%)	61 033 (50.20%)	4 374 (50.98%)	<.001
Diabetes	4 024 (6.61%)	8 086 (6.60%)	1 580 (18.22%)	<.001
Hypertension	39 065 (64.21%)	84 770 (69.22%)	7 272 (83.88%)	<.001
Heart disease	43 (0.07%)	203 (0.17%)	116 (1.34%)	<.001
*APOE* ɛ4 carrier	14 977 (29.35%)	28 499 (27.93%)	1 859 (26.22%)	<.001
uACR (abnormal)	3 379 (18.82%)	6 909 (16.75%)	1 573 (34.82%)	<.001
Brain MRI volume				
Total brain (×10^6^ mm^3^)	1.13 ± 0.11	1.13 ± 0.11	1.11 ± 0.10	<.001
White matter (×10^5^ mm^3^)	5.39 ± 0.61	5.38 ± 0.61	5.33 ± 0.62	.108
Gray matter (×10^5^ mm^3^)	5.95 ± 0.52	5.90 ± 0.52	5.73 ± 0.47	<.001
Hippocampus (×10^3^ mm^3^)	7.38 ± 0.86	7.32 ± 0.87	7.15 ± 0.80	<.001
WMH (×10^3^ mm^3^)	5.07 (2.74, 9.42)	5.29 (2.87, 10.12)	6.22 (3.06, 11.49)	<.001

*Notes*: *APOE* = apolipoprotein E; BMI = body mass index; eGFR = estimated glomerular filtration rate; uACR = urine albumin:creatinine ratio.

Data are presented as mean ± standard deviation, *n* (%) or median (interquartile range).

Missing data: 159 for socioeconomic status, 610 for BMI, 2 761 for education, 876 for race, 1 128 for smoking, 399 for alcohol consumption, 19 522 for physical activity, 1 337 for social connection, 31 792 for *APOE* ɛ4, and 128 247 for uACR.

Linear regression was used to estimate the β-coefficients and 95% CIs for the relationship between kidney function and brain volumes. Besides the confounders mentioned above, these models were additionally adjusted for head size, head position (using x-, y-, and z-axis coordinates), and table position.

Stratified analyses were performed to explore the role of diabetes, hypertension, and *APOE* genotype in the association of kidney function with incident dementia and MRI parameters. To assess multiplicative interactions, we included the cross-product term of kidney function and the variable of interest in the model (ie, kidney × diabetes, kidney function × hypertension, or kidney function × *APOE* genotype).

In sensitivity analysis, we repeated the analyses of the association between kidney function and dementia after (1) using CKD-EPI Creatinine Equation, CKD-EPI 2021 Race-Free Equation, and EKFC Equation to calculate eGFR, (2) additionally adjusting for uACR, and (3) performing multiple imputations for missing values of some covariates.

All *p* values were 2-sided, and we defined statistical significance as *p* < .05. Multiple comparisons were corrected using the false discovery rate (FDR). All statistical analyses were performed using Stata SE 16.0 (StataCorp LLC, College Station, TX) and R (version 4.1.1).

## Results

### Characteristics of the Study Population

Among the 191 970 dementia-free participants, 53.1% were female, with a mean age of 64.1 ± 2.9 years and a mean eGFR of 83.19 ± 12.87 mL/min/1.73 m^2^. Of the participants, 60 837 (31.7%) had normal kidney function, 122 463 (63.8%) had mildly impaired kidney function, and 8 670 (4.5%) had moderately to severely impaired kidney function. Compared to participants with normal kidney function, those with moderately to severely impaired kidney function were more likely to be older, female, White, have a lower education level, have a higher BMI, be a current smoker or drinker, have lower levels of physical activity, have higher levels of social activity, be noncarriers of the *APOE* ɛ4 allele, have abnormal uACR, and have a higher prevalence of diabetes, hypertension, heart disease (*p* value < .05 for all; Table 1). Regarding MRI parameters, those with normal kidney function had on average 2.8 × 10^4^ mm^3^ higher total brain volume, 2.2 × 10^4^ mm^3^ higher gray matter volume, and 2.3 × 10^4^ mm^3^ higher hippocampus volume compared to participants with moderately to severely impaired kidney function. Additionally, the median WMH volume was 1.15 × 10^3^ mm^3^ lower compared to those with moderately to severely impaired kidney function (Table 1).

### Association Between Kidney Function and Dementia

During the follow-up (median [interquartile range]: 12.8 [11.9–13.5] years), 5 327 (2.77%) participants developed dementia, including 2 448 (1.28%) with AD and 1 176 (0.61%) with vascular dementia. In multiply-adjusted Cox regression models, lower eGFR (as a continuous variable; per 1-*SD* decrease) was dose-dependently associated with an increased risk (HR, 95% CI) of dementia (1.10, 95% CI: 1.07 to 1.14), AD (1.06, 95% CI: 1.01 to 1.12), and vascular dementia (1.13, 95% CI: 1.06 to 1.21). Compared to normal kidney function, moderately to severely impaired kidney function was associated with dementia (1.53, 95% CI: 1.32 to 1.76), AD (1.27, 95% CI: 1.00 to 1.60), and vascular dementia (1.51, 95% CI: 1.13 to 2.02). However, mildly impaired kidney function was not significantly associated with dementia, AD, or vascular dementia compared to normal kidney function (Table 2).

**Table 2.** Hazard Ratios (HRs) and 95% Confidence Intervals (CIs) for the Association Between Kidney Function and Subsequent Dementia

Kidney Function	Dementia		AD		Vascular Dementia	
	HR (95% CI)^†^	HR (95% CI)^‡^	HR (95% CI)^†^	HR (95% CI)^‡^	HR (95% CI)^†^	HR (95% CI)^‡^
Continuous (per 1-*SD* decrement)	1.10 (1.07, 1.14)	1.10 (1.07, 1.14)	1.03 (0.98, 1.07)	1.06 (1.01, 1.12)	1.17 (1.11, 1.24)	1.13 (1.06, 1.21)
Categorical						
Normal	1.00 (Ref)	1.00 (Ref)	1.00 (Ref)	1.00 (Ref)	1.00 (Ref)	1.00 (Ref)
Mildly impaired	1.00 (0.93, 1.07)	1.04 (0.96, 1.12)	0.94 (0.85, 1.02)	1.02 (0.91, 1.14)	0.98 (0.86, 1.12)	1.05 (0.89, 1.24)
Moderately to severely impaired	1.61 (1.40, 1.86)	1.53 (1.32, 1.76)	1.14 (0.95, 1.37)	1.27 (1.00, 1.60)^*^	1.92 (1.53, 2.40)	1.51 (1.13, 2.02)

*Notes*: AD = Alzheimer’s disease; CI = confidence interval; HR = hazard ratio; *SD* = standard deviation.

^*^ *p* = .046.

^†^Adjusted for age, sex, and education.

^‡^Adjusted for age, sex, race, socioeconomic status, education, body mass index, smoking status, alcohol consumption, physical activity, social connection, diabetes, hypertension, heart disease, and apolipoprotein E ε4.

In multiply-adjusted Laplace regression models, the 10th PDs (95% CI) of dementia/vascular dementia onset was 1.53 (95% CI: 0.98 to 2.08)/1.40 (95% CI: 0.39 to 2.40) years shorter among participants with moderate to severe impaired kidney function than those with normal kidney function but not significant of AD onset (Table 3).

**Table 3.** 10th Percentile Differences (PDs) in Years of Dementia Onset and 95% Confidence Intervals (CIs) in Relation to Kidney Function

Kidney Function	Dementia		AD		Vascular Dementia	
	10th PD^*^ (95% CI)	10th PD^†^ (95% CI)	10th PD^*^ (95% CI)	10th PD^†^ (95% CI)	10th PD^*^ (95% CI)	10th PD^†^ (95% CI)
Continuous (per 1-*SD* decrement)	−0.39 (−0.49, −0.28)	−0.34 (−0.47, −0.21)	−0.09 (−0.24, 0.05)	−0.21 (−0.39, −0.02)	−0.55 (−0.77, −0.33)	−0.43 (−0.68, −0.18)
Categorical						
Normal	0.00 (Ref)	0.00 (Ref)	0.00 (Ref)	0.00 (Ref)	0.00 (Ref)	0.00 (Ref)
Mildly impaired	−0.02 (−0.24, 0.20)	−0.10 (−0.38, 0.17)	0.23 (−0.08, 0.54)	−0.07 (−0.46, 0.32)	0.05 (−0.41, 0.51)	−0.17 (−0.73, 0.39)
Moderately to severely impaired	−1.75 (−2.16, −1.34)	−1.53 (−2.08, −0.98)	−0.48 (−1.11, 0.16)	−0.77 (−1.62, 0.07)	−2.23 (−3.00, −1.47)	−1.40 (−2.40, −0.39)

*Notes*: AD = Alzheimer’s disease; CI = confidence interval; HR = hazard ratio; *SD* = standard deviation.

^*^Adjusted for age, sex, and education.

^†^Adjusted for age, sex, race, socioeconomic status, education, body mass index, smoking status, alcohol consumption, physical activity, social connection, diabetes, hypertension, heart disease, and apolipoprotein E ε4.

The kidney function–dementia association remained significant after stratification by diabetes status (yes vs no), hypertension status (yes vs no), and *APOE* genotype (ε4 allele carriers vs noncarriers). Furthermore, we found no significant additive or multiplicative interactions between these factors and kidney function on the risk of dementia (Supplementary Table 4).

### Association Between Kidney Function and Regional Brain Volumes

In the multiply-adjusted linear regression, compared to normal kidney function, moderately to severely impaired kidney function was related to a significantly lower gray matter volume (β = -0.11, 95% CI: -0.19 to -0.03, FDR q-value = 0.035); no other brain structural parameters differed significantly between the groups (Table 4). Results from the basic-adjusted models (including only age, sex, education, and head position as covariates) were consistent with those from the multiply-adjusted models (Supplementary Table 5).

**Table 4.** Standardized β Coefficient and 95% Confidence Interval (CI) for the Association of Kidney Function with Structural Brain Volumes

Kidney Function	Total Brain	White Matter	Gray Matter	Hippocampus	WMH
	β (95% CI)	β (95% CI)	β (95% CI)	β (95% CI)	β (95% CI)
Continuous (per 1-*SD* decrement)	0.00 (−0.01, 0.01)	0.01 (−0.00, 0.02)	−0.01 (−0.02, 0.00)	−0.01 (−0.03, 0.01)	−0.00 (−0.02, 0.02)
Categorical					
Normal	0.00 (Ref)	0.00 (Ref)	0.00 (Ref)	0.00 (Ref)	0.00 (Ref)
Mildly impaired	0.01 (−0.01, 0.02)	0.01 (−0.01, 0.03)	−0.00 (−0.02, 0.02)	−0.00 (−0.04, 0.03)	0.01 (−0.03, 0.05)
Moderately to severely impaired	−0.01 (−0.08, 0.05)	0.07 (−0.00, 0.15)	−0.11 (−0.19, −0.03)^*^	−0.10 (−0.24, 0.03)	0.02 (−0.11, 0.16)

*Notes*: CI = confidence interval; MRI = magnetic resonance imaging; *SD* = standard deviation; WMH = White matter hyperintensities.

^*^FDR-adjusted *q* = 0.035.

Adjusted for age, sex, race, socioeconomic status, education, body mass index, smoking status, alcohol consumption, physical activity, social connection, diabetes, hypertension, heart disease, apolipoprotein E ε4, and head position MRI confounds (volumetric data are also corrected for head size).

In stratified analyses, the magnitude and direction of the association between kidney function and gray matter volume remained similar regardless of diabetes status, hypertension status, and *APOE* genotype, but the associations became nonsignificant in each stratum. Again, there was no significant interaction between kidney function and diabetes, hypertension, or *APOE* genotype on gray matter volume (Supplementary Table 6).

### Supplementary Analyses

The associations of kidney function with dementia and regional brain volumes were not much altered when we repeated the analyses after (1) using CKD-EPI 2009 Creatinine Equation, CKD-EPI 2021 Race-Free Equation, and EKFC Equation to calculate eGFR (Supplementary Table 7), (2) additionally adjusting for uACR (Supplementary Table 8), and (3) performing multiple imputations for missing values of some covariates (*n* = 23 639 [12.31%], Supplementary Table 9).

## Discussion

In this large population-based cohort study with an embedded MRI study, we found that moderately to severely impaired kidney function was associated with a higher risk of dementia, including both AD and vascular dementia, and anticipated dementia onset by more than 1.5 years. Moreover, moderately to severely impaired kidney function was related to significantly lower gray matter volume, suggesting that neurodegeneration in the brain might play an important role in the kidney function–dementia association.

Several longitudinal studies have linked impaired kidney function (defined by eGFR) with an increased risk of cognitive impairment and dementia (6–11). Two longitudinal studies reported an association between impaired kidney function and a higher risk of all-cause dementia, AD, and vascular dementia, with a stronger association for vascular dementia than for AD (9,10). Another 2 cohort studies which excluded participants with stroke at baseline also found that participants with lower eGFR have a greater risk of developing dementia than those with normal kidney function (6,8). By contrast, some cohort studies reported no significant association between baseline kidney function and dementia (12–15). One of these studies reported an association between eGFR decline and risk of vascular dementia only (12), while another study included younger participants (mean age 49.5 years) with better kidney function (mean eGFR 78.8 ml/min/1.73 m^2^) (15). A recently published study combining a cohort study, meta-analysis, and Mendelian randomization analyses found no association of impaired kidney function with the risk of dementia in the cohort study or in Mendelian randomization analyses, while in the meta-analysis there was a trend toward increasing estimates for decreasing eGFR for all-cause dementia, but not for AD (11). In the present study, we found that moderately to severely impaired kidney function was associated with an increased risk of dementia, AD, and vascular dementia. More evidence is warranted to reveal whether there is a causal relationship between impaired kidney function and dementia.

Structural brain MRI measures regional brain volume, which could reflect different brain pathological changes and allow for quantitative analysis of specific brain atrophy patterns (38). Decreased gray matter or hippocampus volume is a typical sign of neurodegeneration in the brain (39), while decreased white matter volume (40) or increased WMH lesions are indicators of microvascular lesions (38). Atrophy of the total brain indicates both neurodegenerative and cerebral vascular pathologies (38). So far, previous studies on kidney function and brain structural differences have shown inconsistent results. Several cross-sectional studies have suggested that lower eGFR was associated with a higher WMH burden (17–19), while others showed no such association (21,22). One cross-sectional study with 484 participants aged 60–90 reported that eGFR was not associated with gray matter volume or lobar white matter volume but was strongly associated with deep white matter volume (20). However, another cross-sectional study (*n* = 193; people aged ≥90 years) found an association between low eGFR and lower gray matter volume, but not white matter volume (21). Of 2 previous longitudinal studies on kidney function and brain structural differences—one following 2 671 adults aged over 70 for 5 years (24) and 1 involving 665 adults aged ≥50 years with hypertension and normoglycemia from a randomized controlled trial (23)—neither found an association between lower eGFR and greater WMH burden. The discrepancies among these studies might be explained by relatively small sample sizes, clinical characteristics of the study populations (eg, people with hypertension or cardiovascular disease (17,22,23)), age of the study population (eg, oldest-old individuals (21)), different eGFR distributions (2.1% of moderately to severely impaired kidney function in the analysis of MRI, much lower than in other studies), and different inclusion criteria (not excluding participants with prevalent chronic brain disorders (18–24)). Additionally, many of these studies focused on restricted brain regions or only considered cerebrovascular-related imaging (such as infarcts, cerebral microbleeds, and WMH). In the present study, based on a large population-based cohort of dementia- and neurological disorder-free older adults, we observed that moderately to severely impaired kidney function was related to significantly lower gray matter volume, but not to differences in any other regional brain volumes.

Impaired kidney function disrupts normal body homeostasis and can have a direct detrimental effect on the central nervous system (41). The mechanisms underlying this include accumulating uremic toxins, elevated oxidative stress levels, increased circulating inflammatory factors, impaired blood–brain-barrier integrity, neurotransmitter dysregulation, and disrupted drug pharmacokinetics (41–44). Impaired kidney function may contribute to neurodegenerative changes in the brain by affecting the clearance of perivascular, cerebral, and circulating beta-amyloid (45). Notably, neurodegenerative and microvascular pathologies share common mechanisms and have a reciprocal relationship (16). Indeed, in this study, we found that impaired kidney function was associated with both AD and vascular dementia. However, on MRI, impaired kidney function was associated only with lower gray matter volume (an indicator of neurodegeneration), not with greater WMH volume (an indicator of vascular pathology). The non-significant association between kidney function and WMH might be due to the fact that participants with stroke were excluded from the MRI substudy. Therefore, we speculate that kidney function dysfunction might lead to dementia starting with neurodegeneration followed by vascular damage in the brain. Further studies with longitudinal brain MRI data are warranted to clarify the mechanisms underlying the kidney function–dementia association.

Strengths of this study include the use of a community-based cohort study with large sample size and long follow-up time. In addition, the UK Biobank provided image-derived phenotypes of various brain measures, contributing to a better understanding of the association between kidney function and dementing disorders. Nonetheless, some limitations should be pointed out. First, the participants in the UK Biobank were volunteers and healthier than the general population. Moreover, those with chronic brain disorders such as stroke, brain hemorrhage, and transient ischemic attack were not included in the MRI subsample. This might have led to an underestimation of the association between kidney function and dementia and regional brain volumes, particularly vascular lesions (including reductions in white matter volume and WMH burden), and it may limit the generalization of our findings to other populations (46). Second, the diagnoses recorded in the patient registry have high specificity but relatively low sensitivity (the positive predictive value of dementia cases is 80%–87% in the UK Biobank) (47), and this might also lead to the underestimation of the association between kidney function and dementia risk. However, the observed association in this study is comparable with that reported in our previous study, in which dementia was diagnosed based on yearly follow-up examinations by a physician (eGFR ≥ 60 vs <60; HR = 1.67, 95%CI: 1.14 to 2.44) (48). Therefore, the underestimation may not be substantial. Third, the associations between impaired kidney function and brain MRI measures were estimated based on a cross-sectional design. Though the MRI was collected 9 years after baseline, temporality is not clear due to the lack of repeated MRI measures. Finally, we could not separately analyze the associations between uACR as an additional measurement of kidney function and dementia or structural brain due to missing data. However, the results were not much changed when we added uACR as an additional covariate in the supplementary analysis.

This study provides evidence that poor kidney function is associated with an increased risk of dementia and suggests that neurodegeneration in the brain might play an important role in this association. Our results highlight that maintaining normal kidney function in older age may be a strategy for preventing or postponing the onset of dementia. More evidence is warranted to reveal whether there is a causal relationship between impaired kidney function and dementia.

## Supplementary Material

glad192_suppl_Supplementary_Tables
