# Supplementary material for: Association of Kidney Function With Dementia and Structural Brain Differences: A Large Population-Based Cohort Study
Source: J Gerontol A Biol Sci Med Sci. 2023 Aug 14;79(1):glad192. doi: 10.1093/gerona/glad192 (PMC10733178; doi:10.1093/gerona/glad192)
Supplement: glad192_suppl_Supplementary_Tables [file glad192_suppl_supplementary_tables.docx]

| **Self-reported illness** | **Code (Field ID 20001 and 20002)** | **Number at baseline** |
| --- | --- | --- |
| Dementia or Alzheimer’s disease | 1263 | 178 |
| Parkinson’s disease | 1262 | 858 |
| Chronic degenerative neurological | 1258 | 173 |
| Guillain-Barré syndrome | 1256 | 198 |
| Multiple Sclerosis | 1261 | 1,782 |
| Other demyelinating diseases | 1397 | 74 |
| Stroke or ischemic stroke | 1081 | 6,752 |
| Brain cancer | 1032 | 230 |
| Brain hemorrhage | 1491 | 183 |
| Brain/intracranial abscess | 1245 | 74 |
| Cerebral aneurysm | 1425 | 334 |
| Cerebral palsy | 1433 | 160 |
| Encephalitis | 1246 | 314 |
| Epilepsy | 1264 | 4,061 |
| Head injury | 1266 | 1,625 |
| Infections of the nervous system | 1244 | 54 |
| Ischemic stroke | 1583 | 29 |
| Meningeal cancer | 1031 | 37 |
| Meningioma (benign) | 1659 | 121 |
| Meningitis | 1247 | 2,002 |
| Motor Neuron Disease | 1259 | 56 |
| Neurological injury/trauma | 1240 | 122 |
| Spina bifida | 1524 | 72 |
| Subdural hematoma | 1083 | 196 |
| Subarachnoid hemorrhage | 1086 | 462 |
| Transient ischemic attack | 1082 | 1,872 |
| Neuroma (benign) | 1683 | 159 |
| Other neurological problem | 1434 | 2,010 |

**Supplementary Table 1.** Self-reported chronic brain disorders used as exclusion criteria.

| **Variable** | **Code** |
| --- | --- |
| Age | 21003 |
| Sex | 31 |
| Education | 6138 |
| Townsend deprivation index | 189 |
| BMI | 21001 |
| Race | 21000 |
| Smoking Status | 20116 |
| Alcohol consumption status | 20117 |
| Physical activity | 22032; 894; 914; 884; 904 |
| Social connection | 1031 |
| Hypertension | Field ID 20002: 1065, 1072; 6177, 6153 (medication); 2966; 6150; 4080 (Systolic blood pressure); 4079 (Diastolic blood pressure); 131286 (ICD) |
| Diabetes | Field ID 20002: 1220, 1222, 1223; 2443; 2976; 30750 (HbA1c); 30740 (Glucose); 6153, 6177 (medication); 130706 (ICD) |
| Heart disease | Field ID 20002: 1074, 1075; 6150; 3627, 3894, 131297 (ICD, angina); 131350 (ICD, atrial fibrillation); 131354 (ICD, heart failure) |
| End-stage renal disease | 42026 |
| *APOE* ε4 | rs428358; rs7412 |
| Urinary albumin | 30500 |
| Urinary creatinine | 30510 |
| Serum creatinine | 30700 |
| Cystatin C | 30720 |
| Head size | 25000 |
| MRI data |  |
| Total Brain Volume | 25010 |
| Total Gray Matter | 25006 |
| Total White Matter | 25008 |
| White matter hyperintensities | 25781 |
| Hippocampus (L+R) | 25019/20 |
| Scanner Position X | 25756 |
| Scanner Position Y | 25757 |
| Scanner Position Z | 25758 |
| Scanner Position | 25759 |
| Assessment center | Field ID 54: 11025, 11026, 11027, 11028 |

**Supplementary Table 2.** UK Biobank field codes for all variables used in the current study.

| **ICD Codes** | **Text** | **Alzheimer’s disease** | **Vascular dementia** | **Dementia** |
| --- | --- | --- | --- | --- |
| **ICD-9** |  |  |  |  |
| 290.2 | Senile dementia, depressed or paranoid type |  |  | 🗸 |
| 290.3 | Senile dementia with acute confusional state |  |  | 🗸 |
| 290.4 | Arteriosclerotic dementia |  | 🗸 | 🗸 |
| 291.2 | Other alcoholic dementia |  |  | 🗸 |
| 294.1 | Dementia in other conditions classified elsewhere |  |  | 🗸 |
| 331.0 | Alzheimer's disease | 🗸 |  | 🗸 |
| 331.1 | Pick's disease |  |  | 🗸 |
| 331.2 | Senile degeneration of brain |  |  | 🗸 |
| 331.5 | Creutzfeldt-Jakob disease |  |  | 🗸 |
| **ICD-10** |  |  |  |  |
| A81.0 | Sporadic Creutzfeldt-Jakob disease |  |  | 🗸 |
| F00 | Dementia in Alzheimer's disease | 🗸 |  | 🗸 |
| F00.0 | Dementia in Alzheimer's disease with early onset | 🗸 |  | 🗸 |
| F00.1 | Dementia in Alzheimer's disease with late onset | 🗸 |  | 🗸 |
| F00.2 | Dementia in Alzheimer's disease, atypical or mixed type | 🗸 |  | 🗸 |
| F00.9 | Dementia in Alzheimer's disease, unspecified | 🗸 |  | 🗸 |
| F01 | Vascular dementia |  | 🗸 | 🗸 |
| F01.0 | Vascular dementia of acute onset |  | 🗸 | 🗸 |
| F01.1 | Multi-infarct dementia |  | 🗸 | 🗸 |
| F01.2 | Subcortical vascular dementia |  | 🗸 | 🗸 |
| F01.3 | Mixed cortical and sub-cortical vascular dementia |  | 🗸 | 🗸 |
| F01.8 | Other vascular dementia |  | 🗸 | 🗸 |
| F01.9 | Vascular dementia, unspecified |  | 🗸 | 🗸 |
| F02 | Dementia in other diseases classified elsewhere |  |  | 🗸 |
| F02.0 | Dementia in Picks disease |  |  | 🗸 |
| F02.1 | Dementia in Creutzfeldt-Jacob disease |  |  | 🗸 |
| F02.2 | Dementia in Huntington’s disease |  |  | 🗸 |
| F02.3 | Dementia in Parkinson’s disease |  |  | 🗸 |
| F02.4 | Dementia in HIV disease |  |  | 🗸 |
| F02.8 | Dementia in other specified diseases classified elsewhere |  |  | 🗸 |
| F03 | Unspecified dementia |  |  | 🗸 |
| F05.1 | Delirium superimposed on dementia |  |  | 🗸 |
| F10.6 | Mental and behavioural disorders due to use of alcohol-amnesic syndrome |  |  | 🗸 |
| G30 | Alzheimer’s disease | 🗸 |  | 🗸 |
| G30.0 | Alzheimer’s disease with early onset | 🗸 |  | 🗸 |
| G30.1 | Alzheimer’s disease with late onset | 🗸 |  | 🗸 |
| G30.8 | Other Alzheimer's disease | 🗸 |  | 🗸 |
| G30.9 | Alzheimer's disease unspecified | 🗸 |  | 🗸 |
| G31.0 | Circumscribed brain atrophy |  |  | 🗸 |
| G31.1 | Senile degeneration of brain |  |  | 🗸 |
| G31.8 | Other specified degenerative diseases of nervous system |  |  | 🗸 |
| I67.3 | Binswanger’s disease |  | 🗸 |  |

**Supplementary Table 3.** International Classification of Diseases (ICD) code list for dementia.

**Supplementary Table 4.** Hazard ratios (HRs) of dementia and 10th percentile differences (PDs) of dementia onset in years in relation to kidney function: stratified by diabetes, hypertension, and *APOE* genotype.

| **Kidney function** | **Diabetes** | **Diabetes-free** | **Hypertension** | **Hypertension-free** | ***APOE* ε4 carrier** | ***APOE* ε4 non-carrier** |
| --- | --- | --- | --- | --- | --- | --- |
| **HR (95% CI)** |  |  |  |  |  |  |
| Continuous  (per 1-SD decrement) | 1.13 (1.04–1.22) | 1.10 (1.06–1.14) | 1.11 (1.07–1.15) | 1.08 (1.00–1.16) | 1.09 (1.05–1.15) | 1.11 (1.06–1.17) |
| Categorical |  |  |  |  |  |  |
| Normal | 1.00 (Ref) | 1.00 (Ref) | 1.00 (Ref) | 1.00 (Ref) | 1.00 (Ref) | 1.00 (Ref) |
| Mildly impaired | 1.20 (0.96–1.50) | 1.02 (0.94–1.10) | 1.04 (0.95–1.13) | 1.03 (0.89–1.20) | 1.05 (0.95–1.16) | 1.02 (0.91–1.14) |
| Moderately to severely impaired | 1.44 (1.04–1.99) | 1.58 (1.35–1.86) | 1.54 (1.32–1.80) | 1.46 (0.99–2.14) * | 1.59 (1.31–1.93) | 1.45 (1.17–1.79) |
| **10th PDs (95% CI)** |  |  |  |  |  |  |
| Continuous  ( per 1-SD decrement) | -0.40 (-0.69– -0.11) | -0.33 (-0.48– -0.19) | -0.35 (-0.50– -0.20) | -0.24 (-0.52–0.04) | -0.33 (-0.52– -0.14) | -0.36 (-0.55– -0.17) |
| Categorical |  |  |  |  |  |  |
| Normal | 0.00 (Ref) | 0.00 (Ref) | 0.00 (Ref) | 0.00 (Ref) | 0.00 (Ref) | 0.00 (Ref) |
| Mildly impaired | -0.59 (-1.38–0.21) | -0.05 (-0.34–0.24) | -0.10 (-0.43–0.23) | -0.13 (-0.63–0.37) | -0.17 (-0.56–0.22) | -0.07 (-0.46–0.32) |
| Moderately to severely impaired | -1.07 (-2.28– -0.14) | -1.72 (-2.36– -1.07) | -1.54 (-2.16– -0.93) | -1.30 (-2.54– -0.06) | -1.71 (-2.51– -0.91) | -1.26 (-2.02– -0.50) |

Notes: CI, confidence interval; *APOE*, apolipoprotein E; SD, standard deviation.

Adjusted for age, sex, race, socioeconomic status, education, body mass index, smoking status, alcohol consumption, physical activity, social connection, hypertension, heart disease, and apolipoprotein E ε4, if applicable.

* *P* = 0.053

**Supplementary Table 5.** Standardized β coefficient and 95% confidence interval (CI) for the association of kidney function with structural brain volumes: (Basic-adjust model).

| **Kidney function** | **Total brain** | **White matter** | **Gray matter** | **Hippocampus** | **WMH** |
| --- | --- | --- | --- | --- | --- |
|  | β (95% CI) | β (95% CI) | β (95% CI) | β (95% CI) | β (95% CI) |
| Continuous  (per 1-SD decrement) | 0.00 (-0.01, 0.01) | 0.01^†^ (0.00, 0.02) | -0.02^†^ (-0.03, -0.01) | -0.01 (-0.03, 0.01) | 0.01 (-0.01, 0.02) |
| Categorical |  |  |  |  |  |
| Normal | 0.00 (Ref) | 0.00 (Ref) | 0.00 (Ref) | 0.00 (Ref) | 0.00 (Ref) |
| Mildly impaired | 0.00 (-0.01, 0.02) | 0.02 (-0.01, 0.03) | -0.01 (-0.03, 0.01) | 0.00 (-0.03, 0.03) | 0.00 (-0.03, 0.04) |
| Moderately to severely impaired | -0.01 (-0.07, 0.04) | 0.08^†^ (0.02, 0.15) | -0.13 (-0.20, -0.06) * | -0.09 (-0.20, 0.03) | 0.09 (-0.03, 0.20) |

Notes: SD, standard deviation; WMH, White matter hyperintensities.

Adjusted for age, sex, education, and head position MRI confounds (volumetric data are also corrected for head size).

* FDR-adjusted *q* value < 0.05.

**Supplementary Table 6.** Standardized β coefficient and 95% confidence interval (CI) for the association of kidney function with gray matter volume: stratified by diabetes, hypertension, and *APOE* genotype.

| **Kidney function** | **Diabetes** | **Diabetes-free** | **Hypertension** | **Hypertension-free** | ***APOE* ε4 carrier** | ***APOE* ε4 non-carrier** |
| --- | --- | --- | --- | --- | --- | --- |
|  | β (95% CI) | β (95% CI) | β (95% CI) | β (95% CI) | β (95% CI) | β (95% CI) |
| Continuous  (per 1-SD decrement) | -0.01  (-0.05, 0.04) | -0.01  (-0.02, 0.01) | -0.00  (-0.02, 0.01) | -0.01  (-0.02, 0.01) | -0.00  (-0.03, 0.02) | -0.01  (-0.02, 0.01) |
| Categorical |  |  |  |  |  |  |
| Normal | 0.00 (Ref) | 0.00 (Ref) | 0.00 (Ref) | 0.00 (Ref) | 0.00 (Ref) | 0.00 (Ref) |
| Mildly impaired | -0.06  (-0.18, 0.06) | 0.00  (-0.02, 0.02) | 0.02  (-0.01, 0.05) | -0.02  (-0.06, 0.01) | -0.01  (-0.05, 0.04) | 0.00  (-0.02, 0.03) |
| Moderately to severely impaired | -0.25  (-0.52, 0.01) | -0.09  (-0.17, -0.00) | -0.10  (-0.19, -0.00) | -0.07  (-0.22, 0.08) | -0.08  (-0.25, 0.09) | -0.10  (-0.19, -0.01) |

Notes: SD, standard deviation; *APOE*, apolipoprotein E.

Adjusted for age, sex, race, socioeconomic status, education, body mass index, smoking status, alcohol consumption, physical activity, social connection, diabetes, hypertension, heart disease, apolipoprotein E ε4, and head position MRI confounds (volumetric data are also corrected for head size), if applicable.

**Supplementary Table 7.** Hazard ratios (HRs) and 50th percentile differences (PDs) in years and 95% confidence intervals (CIs) of dementia in relation to kidney function calculated using CKD-EPI 2009 Creatinine Equation, CKD-EPI 2021 Race-Free Equation, and EKFC Equation.

| **Kidney function** | **No. of participants** | **No. of cases** | **Dementia** | | |
| --- | --- | --- | --- | --- | --- |
|  |  |  | HR (95% CI) ^a^ | HR (95% CI) ^b^ | 50th PDs (95% CI) ^b^ |
| **CKD-EPI 2009 Creatinine Equation** |  |  |  |  |  |
| Continuous  (per 1-SD decrement) | 191,970 | 5,327 | 1.03 (1.01–1.06) | 1.04 (1.00–1.07) * | -0.13 (-0.25– -0.02) |
| Categorical |  |  |  |  |  |
| Normal | 84,855 | 2,190 | 1.00 (Ref) | 1.00 (Ref) | 0.00 (Ref) |
| Mildly impaired | 99,655 | 2,812 | 0.93 (0.88–0.98) | 1.00 (0.93–1.07) | -0.01 (-0.25–0.22) |
| Moderately to severely impaired | 7,430 | 325 | 1.40 (1.24–1.57) | 1.32 (1.14–1.54) | -0.94 (-1.44– -0.44) |
| **CKD-EPI 2021 Race-Free Equation** |  |  |  |  |  |
| Continuous  (per 1-SD decrement) | 191,970 | 5,327 | 1.12 (1.09–1.16) | 1.11 (1.07–1.15) | -0.36 (-0.48– -0.24) |
| Categorical |  |  |  |  |  |
| Normal | 93,112 | 2,240 | 1.00 (Ref) | 1.00 (Ref) | 0.00 (Ref) |
| Mildly impaired | 93,169 | 2,791 | 1.10 (1.04–1.16) | 1.12 (1.05–2.07) | -0.41 (-0.65– -0.17) |
| Moderately to severely impaired | 5,689 | 296 | 1.91 (1.68–2.16) | 1.77 (1.51–2.07) | -1.94 (-2.48– -1.40) |
| **EKFC Equation** |  |  |  |  |  |
| Continuous  (per 1-SD decrement) | 191,970 | 5,327 | 1.06 (1.03–1.09) | 1.05 (1.02–1.09) | -0.19 (-0.32– -0.06) |
| Categorical |  |  |  |  |  |
| Normal | 20,300 | 472 | 1.00 (Ref) | 1.00 (Ref) | 0.00 (Ref) |
| Mildly impaired | 160,536 | 4,454 | 0.91 (0.81–1.01) | 0.99 (0.87–1.12) | 0.02 (-0.42–0.46) |
| Moderately to severely impaired | 11,134 | 472 | 1.27 (1.10–1.46) | 1.28 (1.08–1.52) | -0.86 (-1.44– -0.28) |

Notes: Abbreviations: CKD-EPI, Chronic Kidney Disease Epidemiology Collaboration; EKFC, European Kidney Function Consortium; SD, standard deviation. The mean (SD) values of eGFR calculated using CKD-EPI 2009 Creatinine Equation, CKD-EPI 2021 Race-Free Equation, and EKFC Equation are 85.06 (12.19), 88.38 (13.65), and 79.14 (10.70).

^a^ Adjusted for age, sex, and education.

^b^ Adjusted for age, sex, race, socioeconomic status, education, body mass index, smoking status, alcohol consumption, physical activity, social connection, diabetes, hypertension, heart disease, and apolipoprotein E ε4.

* P=0.024

**Supplementary Table 8.** Hazard ratios (HRs) and 50th percentile differences (PDs) in years and 95% confidence intervals (CIs) of dementia in relation to kidney function: additionally adjusted for uACR.

| **Kidney function** | **No. of participants** | **No. of cases** | **Dementia** | | |
| --- | --- | --- | --- | --- | --- |
|  |  |  | HR (95% CI) ^a^ | HR (95% CI) ^b^ | 50th PDs (95% CI) ^b^ |
| Continuous  (per 1-SD decrement) | 191,970 | 5,327 | 1.13 (1.09–1.18) | 1.10 (1.04–1.15) | -0.31 (-0.48– -0.14) |
| Categorical |  |  |  |  |  |
| Normal | 60,837 | 1,457 | 1.00 (Ref) | 1.00 (Ref) | 0.00 (Ref) |
| Mildly impaired | 122,463 | 3,456 | 1.00 (0.90–1.10) | 1.03 (0.90–1.16) | -0.10 (-0.53–0.33) |
| Moderately to severely impaired | 8,670 | 414 | 1.58 (1.35–1.86) | 1.46 (1.19–1.78) | -1.25 (-1.94– -0.57) |

Notes: uACR, urine albumin: creatinine ratio; SD, standard deviation.

^a^ Adjusted for age, sex, and education.

^b^ Adjusted for age, sex, race, socioeconomic status, education, body mass index, smoking status, alcohol consumption, physical activity, social connection, diabetes, hypertension, heart disease, apolipoprotein E ε4, and uACR.

**Supplementary Table 9.** Hazard ratios (HRs) and 50th percentile differences (PDs) in years and 95% confidence intervals (CIs) of dementia in relation to kidney function: after multiple imputation of covariates.

| **Kidney function** | **No. of participants** | **No. of cases** | **Dementia** | | |
| --- | --- | --- | --- | --- | --- |
|  |  |  | HR (95% CI) ^a^ | HR (95% CI) ^b^ | 50th PDs (95% CI) ^b^ |
| Continuous  (per 1-SD decrement) | 191,970 | 5,327 | 1.11 (1.08–1.15) | 1.11 (1.07–1.14) | -0.35 (-0.37– -0.32) |
| Categorical |  |  |  |  |  |
| Normal | 60,837 | 1,457 | 1.00 (Ref) | 1.00 (Ref) | 0.00 (Ref) |
| Mildly impaired | 122,463 | 3,456 | 1.00 (0.94–1.07) | 1.04 (0.97–1.12) | -0.15 (-0.20– -0.09) |
| Moderately to severely impaired | 8,670 | 414 | 1.63 (1.46–1.83) | 1.54 (1.36–1.75) | -1.47 (-1.57– -1.37) |

Notes: HR, hazard ratio; SD, standard deviation.

^a^ Adjusted for age, sex, and education.

^b^ Adjusted for age, sex, race, socioeconomic status, education, body mass index, smoking status, alcohol consumption, physical activity, social connection, diabetes, hypertension, heart disease, and apolipoprotein E ε4.
